# Photosynthetic parameters of a sedge-grass marsh as a big-leaf: effect of plant species composition

**DOI:** 10.1038/s41598-021-82382-2

**Published:** 2021-02-12

**Authors:** Markéta Mejdová, Jiří Dušek, Lenka Foltýnová, Lenka Macálková, Hana Čížková

**Affiliations:** 1 Global Change Research Institute, Academy of Sciences of the Czech Republic, v. v. i., Bělidla 98/4a, Brno, CZ-603 00 Czech Republic; 2 Faculty of Agriculture, University of South Bohemia, Studentská 1668, CZ-370 05 České Budějovice, Czech Republic

**Keywords:** Ecology, Ecophysiology, Wetlands ecology

## Abstract

The study estimates the parameters of the photosynthesis–irradiance relationship (P_N_/I) of a sedge-grass marsh (Czech Republic, Europe), represented as an active “green” surface—a hypothetical “big-leaf”. Photosynthetic parameters of the “big-leaf” are based on in situ measurements of the leaf P_N_/I curves of the dominant plant species. The non-rectangular hyperbola was selected as the best model for fitting the P_N_/I relationships. The plant species had different parameters of this relationship. The highest light-saturated rate of photosynthesis (A_sat_) was recorded for *Glyceria maxima* and *Acorus calamus* followed by *Carex acuta* and *Phalaris arundinacea.* The lowest A_sat_ was recorded for *Calamagrostis canescens*. The parameters of the P_N_/I relationship were calculated also for different growth periods. The highest A_sat_ was calculated for the spring period followed by the summer and autumn periods. The effect of the species composition of the local plant community on the photosynthetic parameters of the “big-leaf” was addressed by introducing both real (recorded) and hypothetical species compositions corresponding to “wet” and “dry” hydrological conditions. We can conclude that the species composition (or diversity) is essential for reaching a high A_sat_ of the “big-leaf ”representing the sedge-grass marsh in different growth periods.

## Introduction

Carbon cycling and its exchange between primary producers (vegetation) and the atmosphere is a central process directly connected with processes of global climate change^1,2^. Individual processes of carbon exchange and sequestration by the vegetation are studied using different approaches and methods from the theoretical and technical points of view^3,4^. A frequently used approach is the simplification of the whole canopy of primary producers to a hypothetical “big-leaf”, representing the active “green” surface of the ecosystem in the meaning of the inclusion of the leaf and canopy models^5,6^. The “big-leaf” concept is also used in post-processing of eddy covariance data^7^ and is associated with the photosynthesis–irradiance relationship (P_N_/I). The eddy covariance technique measures the net ecosystem exchange (NEE) of CO_2_ as the balance between CO_2_ released by ecosystem respiration (Re) and CO_2_ fixation by photosynthesis. The fundamental photosynthesis–irradiance relationship (P_N_/I) is used for separation of underlying processes such as Re. The P_N_/I curves are fitted to daytime NEE measurements, and respiration is estimated from the intercept of the ordinate^7^. This approach allows assessing photosynthetic parameters of a whole plant community as those of a “big-leaf”, without any knowledge of individual contributions of dominant plant species forming this community. The species richness and diversity are important for long-term ecosystem stability due to the higher probability of species-rich communities to survive unfavourable environmental conditions inducing physiological stress^8–11^. Important in this case is functional diversity of the plants in a community, which affects the functions and services of a given ecosystem^12^. Plants have various adaptations determining how they manage different stressors or survive under physiologically unsuitable conditions^13,14^. Knowledge of the photosynthetic parameters of individual dominant plant species based on in situ measurements makes it possible to determine the functional contributions of these species to the photosynthesis of the whole ecosystem characterised as a green “big-leaf” surface. Variation of the “big-leaf” photosynthesis under varied environmental conditions in relation to species composition (diversity) and functional diversity enables us to describe and understand the respective roles of individual species in the photosynthesis of a whole plant community.

The aims of our study are as follows: (1) individual estimation of the parameters of the photosynthesis–irradiance relationship (P_N_/I curve) of five dominant plant species of the sedge-grass marsh community, based on in situ measurements. (2) Based on these estimates, to upscale the parameters obtained for individual species to the “big-leaf” concept of the sedge-grass marsh ecosystem for each growth period (spring, summer and autumn) and also for the whole growing season.

We expected that the wetland plant species studied will differ in values of their photosynthetic parameters, i.e., apparent quantum use efficiency (α), light-saturated rate of photosynthesis (A_sat_), rate of dark respiration (R_d_) and compensation point (I_comp_). These parameters will change during the individual periods of growth, reflecting changing in situ conditions (e.g., position of water table). Contributions of photosynthesis of individual plant species will differ in different growth periods. These differences show the involvement of individual plant species in carbon cycling in the sedge-grass marsh ecosystem.

## Material and methods

### Plant nomenclature

Plant nomenclature: Kaplan, Z., ed (2019)^15^.

### Site description

The sedge-grass marsh is a part of a large wetland complex (450 ha) called “The Wet Meadows” located near the town of Třeboň, South Bohemia, Czech Republic (Central Europe) at the centre of the Třeboň Basin Biosphere Reserve of the UNESCO Man and the Biosphere Programme^16^. The sedge-grass marsh (426 m a.s.l., 49° 01′ 29″ N, 14° 46′ 13″ E) is situated in the inundation area of a large human-made lake (Rožmberk fishpond, water-surface area about 5 km^2^). Its water level is controlled by a system of ditches, which interconnect the system of human-made shallow lakes (fishponds) in the whole region, and is thus fairly stable throughout the year except flood or fishpond-drawdown periods. The traditional use of the Wet Meadows consisted of their regular mowing. The study site represents the wettest part of this wetland, which used to be mown once a year until the 1950s. The resulting vegetation was formed mainly by tall sedges (*Carex acuta* [syn*. C. gracilis*]*, C. vesicaria*) and wetland grasses (mostly *Calamagrostis canescens*)^17–21^. This community is classified as the association *Caricetum gracilis* Almquist 1929^22,23^. During the last 60 years, after cessation of mowing, a distinct structure of hummocks and hollows has developed. The hummocks are formed mainly by tussocks of *C. acuta*. Accompanying species usually colonize hummock edges and extend down into the hollows only during dry years. *C. canescens* spreads on tops of the hummocks in dry years and thus forms an important part of the *C. acuta*–dominated plant community^24^. The other species include *Glyceria maxima* and *Acorus calamus*, which occur either together with *C. acuta* or form monodominant stands. *Phalaris arundinacea* used to colonize only the banks of drainage ditches and small patches of sandy substrate within continuous stands of tall sedges^25^. Surprisingly it has spread over a large area of the sedge-grass marsh after the extreme and long lasting summer flood of 2002^26^.

### Light-curve measurements

The measurements of P_N_/I curves were conducted during the growing season of 2013, from mid-April to early October at weekly intervals except for early June, when the wetland was flooded. The measurements were designed so as to minimise variation among shoots of a particular species, among leaves of different age on a shoot, and random effects of the environmental conditions. A group of adjacent shoots (of *G. maxima*, *P. arundinacea* and *A. calamus*) or individual tussocks (of *C. acuta* and *C. canescens*) were labelled and then repeatedly used for measurements taken during the whole growing season. On a particular date, two shoots were chosen within a labelled plant and their young but fully developed leaves (2nd–4th youngest leaves) were used for the measurement. Four leaves of *C. acuta* and *C. canescens,* respectively, or two leaves of *G. maxima, P. arundinacea* and *A. calamus*, respectively, were enclosed in the leaf chamber so as to cover its whole area. The measured leaf segments were situated at approximately 3/4 of the distance from the leaf-blade base to its apex. All the leaves originated from the top layer of the canopy and were therefore adapted to full sunlight. One photosynthetic light curve was measured for each species on each sampling date.

The rate of net photosynthesis was measured using an open gas-exchange system (Li-Cor 6400, Li-Cor, Inc., Lincoln, NE, USA) equipped with a modulable light source (6400-02B LED). The rapid light response curves were obtained according to the methodology provided in the LICOR-6400 manual. Gas exchange measurements started with a high PAR flux density (2000 µmol (photon) m^–2^ s^–1^) and successively dropped to 1500, 1000, 600, 400, 200, 100, 30 and 0 μmol (photon) m^–2^ s^–1^. The time period between the successive measurements followed the standard protocol (Manual of Li-6400, Book 1, 4–24). i.e., it was at least 2 min followed by a minimum of 15 s of stability of the output signal. This interval was extended to 4 min after the irradiance was set to 0 μmol (photon) m^–2^ s^–1^, i.e. prior to the measurement of dark respiration. The area of the leaf chamber was 6 cm^2^.

Concentration of CO_2_ was set at 400 µmol of mol (air)^–1^ inside the leaf chamber. The flux of the air entering the leaf chamber was set at 300–400 µmol s^−1^ and was measured under natural light conditions at the beginning of each measurement. In order to minimize its fluctuations, the block temperature was set according to the observed ambient temperature in different periods spring (May), summer (June to August) and autumn (September to October). The gas exchange measurements were restricted to the time period from 07:00 to 11:00 h of Central European time in order to minimize the effects of the afternoon depression of photosynthesis^28^.

### Accessory meteorological and leaf area measurements

**Figure 1:**
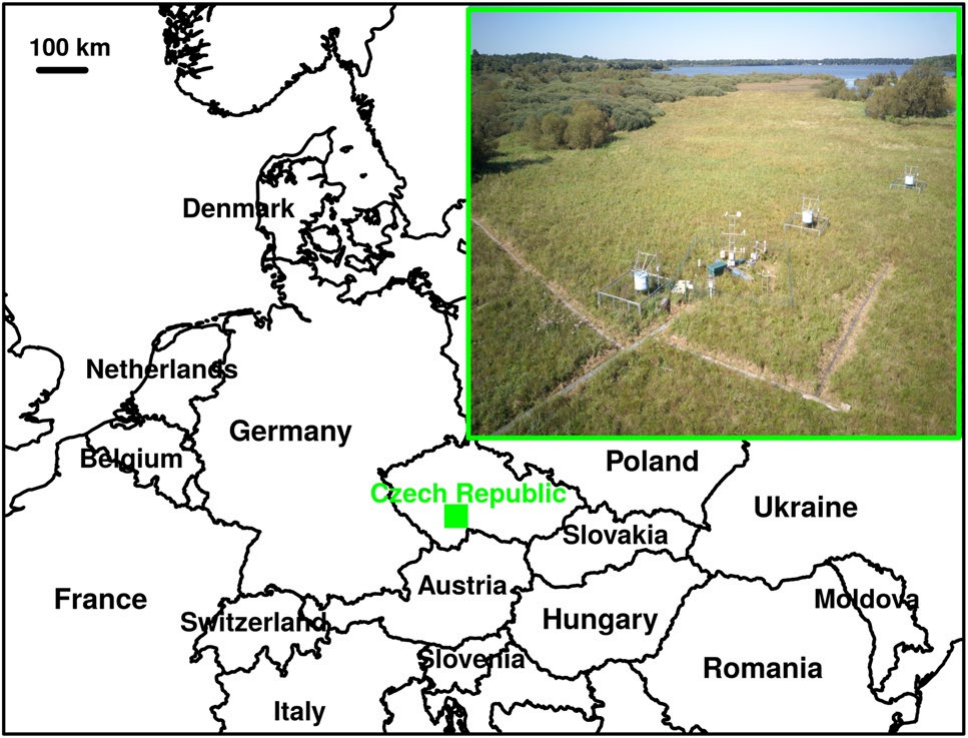
Location of the “Wet Meadows” study site in the Czech Republic (Europe) and picture of the sedge-grass marsh with scientific infrastructure of ICOS (http://www.europe-fluxdata.eu/icos/home) and Fluxnet (http://sites.fluxdata.org/CZ-wet/) flux networks. The map was drawn using the R packages^27^ “rworldmap” version 1.3-6 (https://cran.r-project.org/web/packages/rworldmap/).

**Figure 2:**
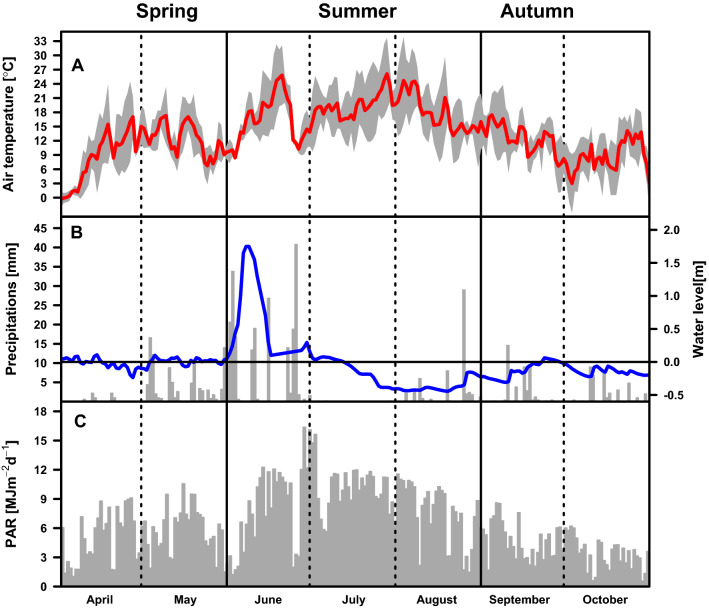
Seasonal courses of (**A**) daily mean air temperature (red line) and its daily fluctuations (grey field), (**B**) daily precipitation (grey vertical bars) and positions of water table (blue line) with (**C**) standard light conditions characterized as daily sums of incident photosynthetically active radiation (PAR) at the sedge-grass marsh.

Meteorological measurements were executed at an ecosystem CZECHWET (CZ-wet) station of ICOS (http://www.europe-fluxdata.eu/icos/home), Fluxnet (http://sites.fluxdata.org/CZ-wet/) and CzeCOS (http://www.czecos.cz/en.html) flux networks. The instruments and sensors are placed on a pontoon which floats during floods (Fig. 1). Photosynthetically active radiation (PAR, sensor EMS Brno, Czech Republic), air temperature (Pt 100, EMS Brno, Czech Republic), water table (LPM-307, BD sensors, Czech Republic) and precipitation (rain gauge Model 376, Met One Instruments, USA) were measured at intervals of 30 s and 30-min averages were saved. Leaf area index (LAI, Fig. 2) was measured using the leaf area meter Li-3100 (Li-Cor, Inc., Lincoln, NE, USA) on 9 occasions during the growing season.

### Photosynthesis–irradiance model and parameters evaluation

In general, photosynthesis or net photosynthesis (P_N_) increases with increasing PAR irradiance (I)^29^. Many different mathematical models can be used for exact description of this relationship (see Supplementary Information for details). Non-rectangular hyperbola was selected as the best model of the P_N_/I relationship in our study^29,30^ on the basis of previous precise analyses of other available models used for describing the P_N_/I relationship (see Supplementary Information for details).

Individual parameters of the photosynthetic models were estimated as follows: apparent maximum quantum yield (α), light compensation point (I_comp_) and the light-saturated rate of net photosynthesis (A_sat_) were modelled using the function nls2 (https://cran.r-project.org/web/packages/nls2/) in the R statistical software version 3.6.1^27^. This function determines the non-linear (weighted) least-square estimates of the parameters of a non-linear model^31^. Dark respiration (R_d_) was estimated as the intersection of the modelled curve with the Y axis. The models were parametrized separately for each species for each measurement day and for each growth period. The parameter of convexity (*θ*) in the non-rectangular hyperbola model was fitted within a fixed interval from 0 to 1 according to Ögren^32^.

Obtained parameters of the P_N_/I relationship were first tested for normality by the Shappiro–Wilk normality test^33^. As they did not have a normal distribution, the non-parametric Kruskal–Wallis test^33^ was used for subsequent evaluation of differences in parameters of photosynthetic light-curve between examined plant species. Post-hoc testing was performed using the Dunn test with Bonferroni correction for multiple comparisons by means of the Kruskal–Wallis test. The relationship between the parameters of environmental conditions was analysed by correlation analysis (Spearman’s nonparametric correlation coefficient^34^).

### The big-leaf photosynthetic parameters

As the most suitable parameter for upscale in situ measurements at leaf level to an ecosystem level the sedge-grass “big-leaf” was used leaf area index (LAI) of studied plant species. Average parameters of the P_N_/I relationships calculated for individual species were weighted by corresponding portions of LAI for different growth periods as well as the whole growing season. The parameters of P_N_/I “big-leaf” relationships was obtained as the sum of individual weighted parameters of the plant species. The final course of the P_N_/I relationship of the sedge-grass “big-leaf” was presented by substituting obtained parameters into the equation of a non-rectangular hyperbola (model 4, Supplementary Information Table S1).

## Results

### Environmental conditions during the growing season

The environmental conditions varied characteristically during the year in accordance with the site location in the temperate zone (Fig. 2). In spring, the daily average amount of incident photosynthetically active radiation (PAR) was 5.4 MJ m^−2^ d^−1^ (maximum PPFD 1645 µmol m^−2^ s^−1^). The highest daily average amount of incident PAR was recorded in summer 8.7 MJ m^−2^ d^−1^ (maximum PPFD 2063 µmol m^−2^ s^−1^). In the autumn the daily average amount of incident PAR was the lowest only 4.1 MJ m^−2^ d^−1^ (maximum PPFD 1558 µmol m^−2^ s^−1^). A similar pattern was found for the daily mean temperature, which was 10.4 °C, 16.6 °C, and 10.7 °C in spring, summer and autumn, respectively.

Precipitation differed among individual periods and months (Fig. 2B). The highest monthly sums of precipitation were recorded in May (109 mm) and June (212 mm). Very low precipitation (less than 1 mm in total) was recorded in July. The low precipitation period persisted also in August (58 mm, which is about half of the long-term average).

Water level fluctuated within periods and also within individual months (Fig. 2B). It remained near the soil surface in spring. A flood (with maximum water level at 1.75 m) occurred in June after several days of extremely high precipitation. As a result of no precipitation in July, the water level sank to 0.44 m below the soil surface and remained there until mid- September. The water level fluctuations were significantly correlated with the precipitation in the spring (R_spearman_ = 0.31, *p* < 0.05) and summer (R_spearman_ = 0.32, *p* < 0.01) periods, but not in the autumn period.

### Vegetation composition and leaf area index

**Figure 3:**
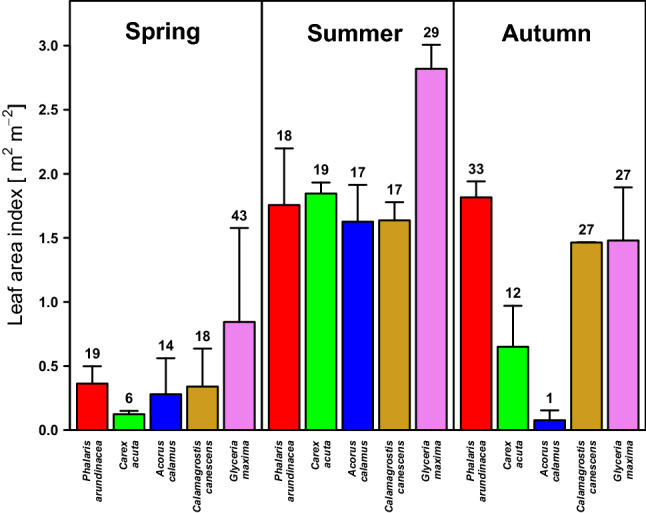
Leaf area index (LAI) of individual dominant plant species of the sedge-grass marsh during different growth periods: spring, summer and autumn. Average LAI values are presented with standard errors of mean (SE). Numbers above the SE-indicating abscissae are percentages of total LAI formed by each of the five dominant plant species in each growth period.

Vegetation of a sedge-grass marsh was without significant changes appearing in changing abundances and species composition during the studied growth season. The relative cover of the dominant species was following: *Phalaris arundinacea* 35%, *Carex acuta* 28.6%, *Glyceria maxima* 16.5%, *Calamagrostis canescens* 18.7% and *Acorus calamus* 1.2%. Different state of plant developing was determined by changes of leaf area index (LAI, Fig. 3) among periods of the growing season. Different LAI among periods and plant species relate to slightly different plant phenology.

LAI increased gradually from the spring (1.95 m^2^ m^−2^) to the summer period, when total LAI reached its maximum of about 9.7 m^2^ m^−2^. During the autumn, LAI decreased to about 5.5 m^2^ m^−2^. LAI values significantly differed between the three growth periods (χ^2^ = 20.6, *p* < 0.05). The shares of individual plant species in total LAI were also different across the growth periods. The highest proportion of total LAI was formed by *Glyceria maxima*: 43% in spring, 29% in summer and 27% in the autumn. During the summer period, LAI values of individual species were quite balanced within the range of 17% to 19% of total LAI, except *Glyceria maxima* (29%). The lowest proportions of total LAI were those of *Carex acuta* and *Acorus calamus* plants in the spring and autumn. In the autumn period, *Phalaris arundinacea* and *Glyceria maxima* formed the highest proportions of total LAI (Fig. 3). For the whole growing season, the relative LAI proportions of the five dominant species were as follows: *P. arundinacea* 23.0%, *C. acuta* 15.3%, *A. calamus* 11.6%, *C. canescens* 20.1% and *G. maxima* 30.0%.

**Figure 4:**
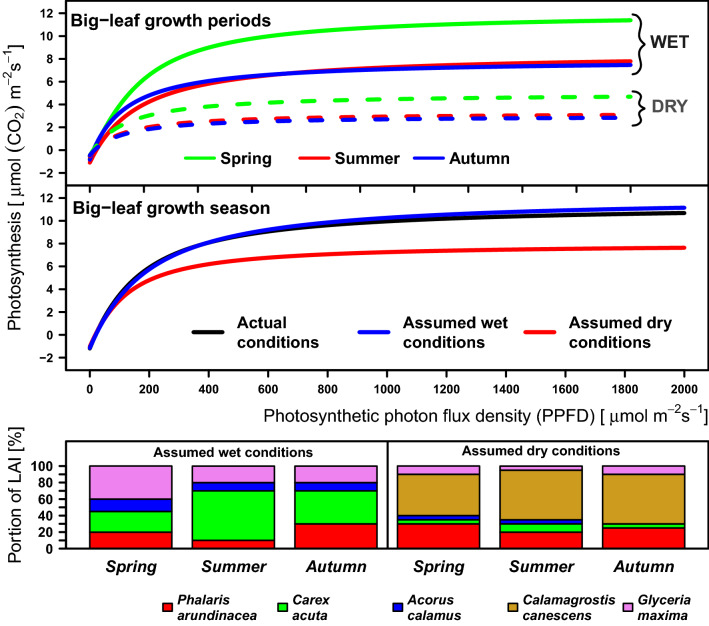
Photosynthesis–irradiance weighted relationship (P_N_/I) of a hypothetical “big-leaf” for the hypothetical “wet” and “dry” hydrological situations and corresponding hypothetical plant species compositions. The PN/I relationships were modelled by non-rectangular hyperbolas for individual growth periods and also for the whole growing season. The proportional bar plot shows the plant species composition (proportions of LAI) in two hypothetical hydrological situations for individual growth periods.

Based on relative proportions of LAI, two hypothetical species composition variants of the plant community were created. They were composed of *Phalaris arundinacea*, *Carex acuta*, *Glyceria maxima*,* Calamagrostis canescens* and *Acorus calamus* (Fig. 4). Two habitat variants corresponding to two hypothetical simple hydrological situations: “wet” and “dry”, were set separately for each growth period, i.e., spring, summer and autumn. The species composition variants (percentage proportions of each species’ LAI Fig. 4) were also set for the “wet” and “dry” hydrological situations. The hypothetical “wet situation is determined by the position of the water level closely below or above the soil surface (up to + 0.4 m), while the “dry” hypothetical situation is determined by the water level situated markedly below the soil surface (maximally sinking to − 0.4 m). At the hypothetical “wet” situation, we assume *Carex acuta* to form the highest LAI proportion during the summer period, with *Glyceria maxima* and *Phalaris arundinacea* forming it during the spring and autumn periods, respectively. *Calamagrostis canescens* is not represented in the hypothetical plant community at the “wet” situation. On the other hand, in the “dry” situation, we assume the highest LAI proportion being formed by *C. canescens* and *P. arundinacea*. Other plant species were represented at minute to negligible hypothetical LAI proportions.

### ***P***_***N***_***/I relationship and individual curve parameters***

**Figure 5:**
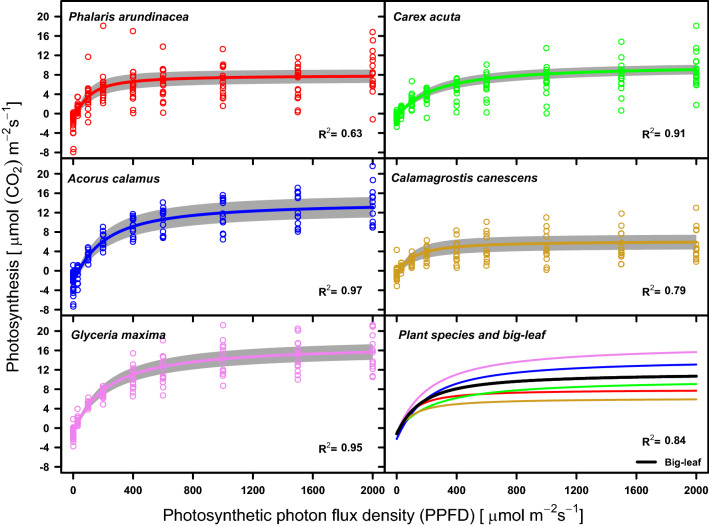
Photosynthesis–irradiance (PPFD) relationship (P_N_/I) of the five plant species and weighted P_N_/I relationship for a theoretical “ecosystem big-leaf” (black line) modelled by non-rectangular hyperbolas. Grey areas represents standard errors of average P_N_/I relationships.

The relationship between photosynthesis and PAR irradiance modelled by a non-rectangular hyperbola (Fig. 5) differs among the plant species studied. They can be divided into three groups according to the A_sat_ curve parameter (Table S5) which determine a position of P_N_/I curve relative to other plant species. The position of curves of the plants, with the highest A_sat_, included *A. calamus* and *G. maxima*, their A_sat_ reaching on average 16.61 µmol m^−2^ s^−1^ and 18.36 µmol m^−2^ s^−1^, respectively were above others. The group comprised *P. arundinacea* and *C. acuta* with average A_sat_ values 9.41 µmol m^−2^ s^−1^ and 10.83 µmol m^−2^ s^−1^, respectively had P_N_/I curves lower. The last species *C. canescens*, with the lowest A_sat_ (A_sat_ = 7.02 µmol m^−2^ s^−1^ in average) was the lowest position of P_N_/I curve from the plant species studied. The differences among species A_sat_ were statistically significant at *p* < 0.05 probability level.

The highest proportion of the variability of P_N_/I relationship explained by the model was calculated for *A. calamus* and *G. maxima* (R^2^ = 0.97 and 0.91). Explained variability of *C. acuta* and *C. canescens*, were lower (R^2^ = 0.91 and 0.79). The lowest variability explained by the P_N_/I model was calculated for *P. arundinacea* (R^2^ = 0.63). The weighted P_N_/I curve of the “big-leaf” explained about 84% of variability of the P_N_/I relationship (Fig. 5).

#### Light-saturated photosynthesis (A_sat_)

**Figure 6:**
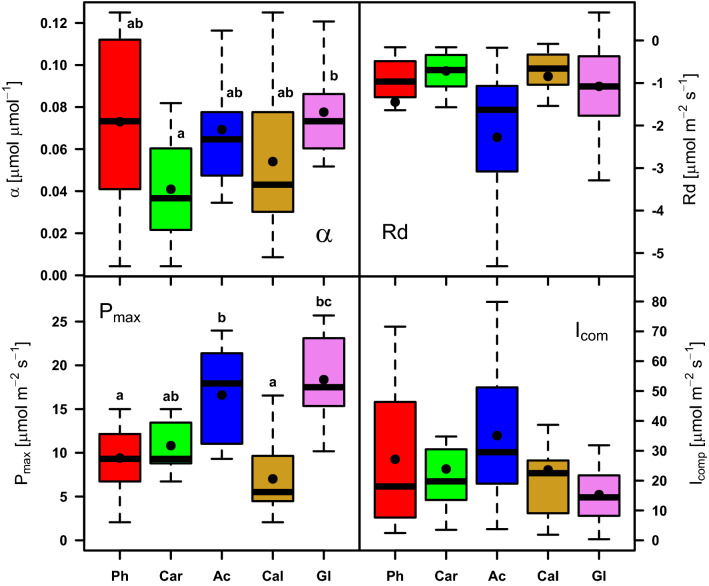
Parameters of the P_N_/I relationships: α apparent maximum quantum yield, Rd- dark respiration, I_com_—irradiance compensation point, A_sat_—light-saturated rate of photosynthesis, of the respective plant species: Ph—*Phalaris arundinacea*, Car—*Carex acuta*, Gl—*Glyceria maxima*, Ac—*Acorus calamus*, Cal—*Calamagrostis canescens*). Mean (black dot) and median (thick line) are shown inside each box. Statistically significant between-species differences of each parameter are indicated by different letters above the respective boxes compared.

The maximum rates of photosynthesis varied considerably among the selected plant species (Fig. 6). The highest values of A_sat_ were found in *G. maxima* and *A. calamus* during May and June (about 26 µmol m^−2^ s^−1^). Their A_sat_ decreased by about 25% from July to the middle of August. And finally, A_sat_ markedly decreased, by about 45%, in the rest of growing season. The photosynthetic rates of *C. acuta* and *P. arundinacea* reached their highest A_sat_ values after the flood (about 20 µmol m^−2^ s^−1^). In the rest of the growing season, A_sat_ decreased and varied between 6 and 13 μmol^−2^ s^−1^. *C. canescens* reached its highest A_sat_ of 17.6 µmol m^−2^ s^−1^ after the July flood and then decreased. A_sat_ of *C. canescens* remained stable after the temperature drop in August and fluctuating around 5 μmol m^−2^ s^−1^. After a rise of the groundwater table in September, A_sat_ increased to about 8 µmol m^−2^ s^−1^.

#### Apparent maximum quantum yield (*α*)

The average values of apparent maximum quantum yield (*α*) ranged from 0.041 µmol CO_2_ µmol (photon)^−1^ in *C. acuta* to 0.078 µmol CO_2_ µmol (photon)^−1^ in *G. maxima*, with average values of the other species in-between. There was only one significant between-species difference, namely that between *C. acuta* and *G. maxima* (*p* < 0.05, Fig. 6). The values of α had a similar seasonal trend in all five plant species studied. They increased after the decrease of air temperature following the flood.

#### Dark respiration rate

Overall dark respiration (R_d_) was higher at the beginning of the growing season and increased also after the flood. R_d_ increased also at the end of June and at the time of dry soil at the beginning of August. A strongly increasing rate of dark respiration was observed towards the end of the growing season. *A. calamus* reached the highest seasonal average R_d_ of all five species studied, but none of the between-species differences was significant. The minimum and also the most stable R_d_ values out of all five species were found for *C. acuta*; by contrast, the most variable R_d_ was that of *P. arundinacea* (Fig. 6).

#### Compensation point (*I*_*comp*_)

The value of *I*_*comp*_ was influenced by the input of photosynthetic photon flux density (PPFD). Higher PPFD resulted in a higher I_comp_. When PPFD was above 1000 µmol (photon) m^−2^ s^−1^ I_comp_ was about 14–19 µmol (photon) m^−2^ s^−1^ and about 20 µmol (photon) m^−2^ s^−1^ for *P. arundinacea* and *C. canescens*, respectively. When PPFD was less than 500 µmol (photon) m^−2^ s^−1^, the I_comp_ was about 6 (photon) µmol m^−2^ s^−1^ for both plant species.

A different trend was observed for *A. calamus.* It reached higher values of I_comp_, of about 50 µmol (photon) m^−2^ s^−1^, during days with high PAR irradiance. With decreasing PPFD during August I_comp_ decreased on average to 27 µmol (photon) m^−2^ s^−1^ and another decrease to 7 µmol (photon) m^−2^ s^−1^ followed in September. No trend of I_comp_ was found for *G. maxima* and *C. acuta*, with their I_comp_ varying between 0.01 and 27.4 μmol (photon) m^−2^ s^−1^ during the growing season. The seasonal averages of the irradiance compensation point (I_comp_) varied between 13.36 μmol (photon) m^−2^ s^−1^ and 22.49 μmol (photon) m^−2^ s^−1^ for the studied species, except for *A. calamus*, which reached 29.52 µmol (photon) m^−2^ s^−1^. The differences among the five plant species were, however, not significant (Fig. 6).

### The P_N_/I relationships at real and hypothetical plant species compositions

**Figure 7:**
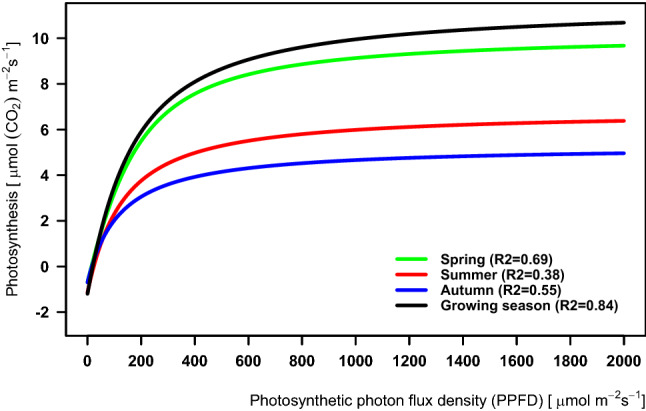
Average photosynthesis–irradiance relationship (P_N_/I) of the theoretical “ecosystem big-leaf” modelled by non-rectangular hyperbolas with real plant species composition. The P_N_/I relationships are presented separately for different periods: spring, summer and autumn. The P_N_/I relationship including the whole growing season is shown for comparison with those for individual growth periods. All curves were weighted by the plant species´ partial LAI values in individual growth periods (Fig. 3).

In a real plant community whose species composition is modelled by a non-rectangular hyperbola, the weighted PN/I relationship is different in each period of the growing season (Fig. 7). The curves are sorted in descending order from the curve for the “big-leaf” in the whole growing season, showing the highest values of light-saturated photosynthesis (A_sat_). The next PN/I curves for the “big-leaf” of the spring, summer and autumn periods followed the curve for the whole growing season with lower maximum values of A_sat_. Most of the differences among the curve parameters for individual periods were not significant. We found only one difference significant at *p* < 0.05 probability level, namely that between the irradiance compensation points (I_comp_) in the summer and autumn periods (Table S4).

The PN/I weighted relationship was different for the two hypothetical plant compositions in the hypothetical “wet” and “dry” hydrological situations (Fig. 4). For all growth periods. Higher A_sat_ was modelled for the “wet” variant as compared with the “dry” variant. Within the “wet” variant, the highest A_sat_ was modelled for the spring period; the other curves are sorted in descending order from the spring one through that for summer to the autumn one. However, in the “dry” variant, the highest A_sat_ was modelled for the summer and autumn periods while the modelled lowest A_sat_ occurred in the spring period. A comparison of whole-season weighted curves for the “big-leaf” shows that the hypothetical “wet” plant species composition variant of the sedge-grass marsh community reaches a higher A_sat_ than the real plant species composition variant. The difference is, however, very small (Fig. 4). Greater differences are evident between the “dry” and “wet” plant species composition variants as well between the “dry” and real variants.

### The plants’ photosynthetic response to the flood

The photosynthetic response to water-table fluctuations was measured in *P. arundinacea*, *C. acuta* and *A. calamus*. Photosynthesis was not measured in *C. canescens* and *G. maxima* before the flood; therefore their respective reactions to flooding cannot be evaluated. A flood (with a maximum water level of 1.75 m) occurred in June 2013 after a period of extremely high precipitation (Fig. 2B). The vegetation was flooded for about 11 days, when the water level was at least 0.5 m above soil surface (height of *C. acuta* tussocks). The water level dropped to the height of the sedge tussocks within three days. At the first measurements after the flood, we recorded an increase of α (*A. arundinacea*, *C. acuta* and *A. calamus*) and increasing absolute values of R_d_ (*A. arundinacea* and *C. acuta*). In some of the first measurements after the flood, A_sat_ either increased (*P. arundinacea* and *C. acuta*), or decreased (*A. calamus*). The flooding did not affect the plants’ photosynthetic parameters measured both immediately and later after the flood.

## Discussion

### Photosynthetic parameters of the studied wetland plant species

#### Maximum photosynthetic capacity

In general, sedges have a higher net assimilation under waterlogged than under drained conditions with a maximum rate of their net assimilation in the range of 10 to 15 μmol m^−2^ s^−1^ ^35^. Ondok and Gloser^36^ reported A_sat_ of *C. acuta* at different leaf temperatures (10, 20 and 30 °C) as 13.63, 15.91 and 17.05 μmol m^−2^ s^−1^, respectively. A_sat_ of *C. acuta* was on average 10.83 μmol m^−2^ s^−1^. This is the third highest average of the five plant species studied. The last two plant species are not strictly wetland ones, being capable of growing in water-saturated organic soil for a long time. *C. canescens* grows mainly on tops of sedge tussocks or on raised sites and *P. arundinacea* grows on the banks of the Central Channel. These plant species showed average A_sat_ 7.02 and 9.41 μmol m^−2^ s^−1^, respectively. Ondok and Gloser^36^ reported Asat of *C. canescens* in range of 7.02 to 11.36 μmol m^−2^ s^−1^. A_sat_ of the moderately flood-tolerant species *P. arundinacea,* ranged from 5 to 7 μmol m^−2^ s^−1^ ^37^ but its A_sat_ measured in container-grown plants was higher: between 12 and 17 μmol m^−2^ s^−1^ ^38^. Slightly smaller values were obtained in a flooding experiment, where A_sat_ of *P. arundinacea* was within the range of 9.9 to 12.5 μmol m^−2^ s^−1^ ^39^. Gloser^40^ reported the net photosynthetic rate of *P. arundinacea* being 11.41 μmol m^−2^ s^−1^. This value is within the range mentioned previously. In our measurements, the A_sat_ was within the range of 2.07 to 15.00 μmol m^−2^ s^−1^.

#### Dark respiration

Dark respiration (R_d_) is related to the developmental stage of the leaves and the period of the growing season^13^. R_d_ depends mainly on leaf temperature^41^ and physiological condition of leaves (mainly water saturation). The typical range of R_d_ values is from − 0.7 to − 2.5 μmol m^−2^ s^−1^ for C_3_ plants and from − 1.4 to − 2.5 μmol m^−2^ s^−1^ for C_4_ plants^42^. Nobel^29^ presents R_d_ derived from a generalized hyperbolic function of C_3_ plants, being about − 1 μmol m^−2^ s^−1^ of CO_2_ at A_sat_ of about 12 μmol m^−2^ s^−1^. A wide range of R_d_ (− 0.41 to − 6.28 μmol m^−2^ s^−1^), obtained in our measurements, is usually due to different shapes of light curves of the plant species studied. Maximum absolute values of R_d_ were recorded for *A. calamus* (− 6.28 μmol m^−2^ s^−1^) and *P. arundinacea* (− 5.94 μmol m^−2^ s^−1^) after the flood and also in October. These R_d_ values are consistent with those obtained for *C. acuta, G. maxima* and *C. canescens* by Ondok and Gloser^36^. R_d_ values similar to those of our selected plant species are also consistent with values reported for other wetland plant species such as *Schoenoplectus hallii:* − 1.3 ± 0.6 μmol m^−2^ s^−1^ ^43^, *Phragmites australis: − *2.62 ± 0.11 μmol m^−2^ s^−1^ and *Carex cinerascens − *1.58 ± 0.09 μmol m^−2^ s^−1^ ^44,45^. Experimentally measured R_d_ in *P. arundinacea* was about − 0.35 μmol m^−2^ s^−1^ in the control treatment and − 0.66 μmol m^−2^ s^−1^ in the submergence treatment^46^.

The apparent R_d_ was surprisingly high after the flood. In principle, this could have been a result of two processes: (1) plant respiration and (2) CH_4_ formation in fermentative processes in anaerobic soil and its subsequent oxidation to CO_2_ when it can pass through a vented aerenchymatous plant tissue. Unfortunately, the methodology used does not allow a quantification of the proportionality of the two processes. Nevertheless, we assume that the amount of CO_2_ vented from the soil was unimportant in this study because the soil was saturated with water (and therefore anaerobic) not only during the high flood pulse, but also before and after it (cf. Fig. 2). If venting of CO_2_ from the soil had been important, then the measured R_d_ would have been similar before and after the flood pulse, but this was not the case. Therefore, we assume that the measured R_d_ reflects mainly increasing respiration rate, indicating resumed growth of aboveground plant parts after their exposure to flooding. Recovery growth requires, among others, energy for transport of storage carbohydrates to the apical meristems, cambium and other growth tissues. This process is also directly reflected in dark respiration^47,48^. Unfortunately, our photosynthesis measurements did not enable us to distinguish the proportion of recorded R_d_ reflecting the intensity of fermentative processes from its proportion representing dark (growth/maintenance) respiration of the plants as such^42^.

#### Apparent maximum quantum yield

The quantum yield α is represented by the initial linear slope of a generalized hyperbolic relationship between incident photosynthetic photon flux density and the rate of net CO_2_ uptake by plants (photosynthetic rate). In C_3_ plants, it is about 0.05 μmol(CO_2_) μmol(photon)^–1^ ^29^. The theoretical maximum value of α is 0.1250 μmol(CO_2_) μmol(photon)^–1^ as 8 photons are required per one molecule of CO_2_ fixed. Determination of a correct real maximum quantum yield must be the nearest one to this theoretical value^49,50^. The value of α has a mathematical definition but it often does not have the desired ecophysiological significance. This vagueness is due to the heterogeneity of methods used for the calculation of this parameter; the results of various calculations of α must therefore be interpreted with caution. Another problem is the curvilinear shape of the P_N_/I relationship throughout its length, and thus the linear phase is not clearly identifiable^51^. In our study, the range of apparent maximum quantum yield ranged from 0.051 to its theoretical maximum value of 0.125 μmol μmol^−1^. The highest average α was calculated for *P. arundinacea* and *A. calamus* in August. The lowest one was calculated for *C. acuta* and *C. canescens*. This finding is consistent with the shape of their light curves and low A_sat_.

#### Light compensation point

The light compensation point (I_comp_) is derived from a generalized hyperbolic relationship between the incident photosynthetic photon flux density and the net CO_2_ uptake rate (photosynthetic rate). In C_3_ plants, it is 15 μmol m^−2^ s^−1^ on the average^29^, within the average range of 8 to16 μmol mol^−2^ s^−1^ ^29^. A comparative review of the genus *Carex*^52^ reported I_comp_ values ranging from 20 to 70 μmol m^−2^ s^−1^. Low I_comp_ values of plants, including sedges, have been reported mainly for herbaceous species occurring in a variable light environment under a deciduous forest canopy^53^. This I_comp_ usually increases from sites below a dense canopy towards canopy gaps, ranging from 4.2 ± 2.1 to 17.0 ± 2.9 μmol m^−2^ s^−1^ ^54^. Smith and Houpis^43^ presented I_comp_ of *Schoenoplectus hallii* of about 24 μmol m^−2^ s^−1^. The I_comp_ of *Phragmites australis* and *Carex cinerascens* were about 48.5 and 37.8 μmol m^−2^ s^−1^, respectively^44^. Flooding usually increased the I_comp_, like the effect of canopy gaps^44,45^.

Our calculated I_comp_ values are within the range reported for *Carex* species^52^ and are highly variable. The range was from 7.35 to 31.84 μmol m^−2^ s^−1^. The lowest I_comp_ was calculated for *C. acuta* and the highest was for *A. calamus*. I_comp_ can characterize plant adaptation to sunny and shady habitats. Leaves acclimated to shade respire less and are less stressed by light than leaves acclimated to high irradiance (sunny leaves). The low I_comp_ of *C. acuta* is related to the structure of its leaves and stand with the leaf bases being self-shaded in sedge tussocks. A contrasting situation develops in stands of *A. calamus*, whose straight leaves shade the leaf bases less than it is the case in *C. acuta*. I_comp_ estimated from the P_N_/I curves of the five selected wetland species is consistent with the ecophysiological principle that I_comp_ is lower in leaves adapted to shade than in leaves exposed to direct sunlight^13^.

### Photosynthesis of the sedge-grass community: effect of the flood

Wetland plants tolerate fluctuating water levels and can also survive submergence during floods. Photosynthesis of emergent plants under water is inhibited due to reduced stomatal conductance^45^, decreased availability of PPFD^55^, high photorespiration^56^ and decrease of chlorophyll content^45^. In our case the flood effect was not significant due to the short duration of the flooding (only 11 days). The plants resumed their growth without visible long-term damage immediately after the flood retreated. Undeveloped wetland plant species can withstand short floods due to their tolerance to flooding^13^. Some species were still without leaves or with only partially developed leaves at the end of spring. The young leaves are usually not damaged at all or very little by a short-time flood. The plants have sufficient carbohydrate reserves^57^ in their belowground parts (stem bases and rhizomes) at this time. They can use them for a—fast recovery of their growth after the short-time flood subsides. Similar results were reported by Li et al.^44^, who analysed the effects of flooding on gas exchange in leaves of *Phragmites australis*, *Carex cinerascens*, and *Hemarthria altissima*.

### Photosynthetic parameters of the big-leaf: effect of plant species composition

Sedge-grass marshes belong to fens with plant communities formed by a mixture of herbaceous plant species dominated by rhizomatous graminoids^58^, which determine the physiological characteristics of the whole fen plant community^36,59^. Different physiological properties of the plant species are usually linked with the plants´ structure creating spatial ecological niches^60^. These niches are closely associated with the physiological functioning of the individual plant species as well as the whole plant community, and vice versa.

Canopy structures of *G. maxima* and *C. acuta* are more effective in the competition for PPFD thanks to their more erectophile foliage than that of *C. canescens*^36^. In *G. maxima,* the greatest proportion of its foliage is concentrated in its higher canopy layers and this species thus tolerates shading. *A. calamus* is a heliophyte with its assimilatory surface placed mainly in its lower canopy layers^61–63^. Leaves of *A. calamus* expand earlier than those of the other species^61^, thus compensating the poorer competitive ability of *A. calamus*^64^.

Based on the above mentioned canopy structure and physiological properties of the *G. maxima* and *A. calamus*, we assume that plant communities with dominant *G. maxima* and/or *A. calamus* reach higher A_sat_ especially under optimal hydrological situations. This assumption is confirmed by the real species composition of the plant community, and also by the hypothetical “wet” variant of its species composition. The “wet” variant reached A_sat_ of 13.27 μmol m^−2^ s^−1^, which is close to the lower values of A_sat_ in the real in situ plant species composition variant (Fig. 4).

The P_N_/I relationship of the “big-leaf” was different in individual growth periods (Fig. 7). The comparison of these periods shows a relatively large difference between the spring and the other periods in both the real and the “wet” variants. The highest A_sat_ of the P_N_/I relationship during spring was predictable on the basis of the P_N_/I relationship of *A. calamus,* a heliophyte^64^ which is photosynthetically highly active in spring, when its growth starts early and its leaves are not shaded by other dominant plant species^61,62,64^. Higher achieved A_sat_ also corresponds to the higher LAI proportion of *Glyceria maxima* and *Acorus calamus* in both the real variant and the “wet” one during the spring period.

The sedge *C. acuta* is a species which is characteristic of the studied fen community. Its PN/I relationship curve is very similar to that of the whole growing season´s P_N_/I relationship of the “big-leaf” derived from the real species composition of the Wet Meadows´ plant community (Fig. 5). *C. acuta* is thus a keystone species creating the unique spatial structure of the sedge-grass marsh by forming its hummocks and hollows structure^59^. If we reduce the LAI proportion of *C. acuta* together with that of such species as *G. maxima* and *A. calamus* in favour of *C. canescens,* which we assume to occur in the “dry” variant of plant species composition, the P_N_/I relationship reaches lower A_sat_ values for individual growth periods (5.457 to 7.862 μmol m^−2^ s^−1^ on the average) and also for the whole growing season: A_sat_ = 9.079 μmol m^−2^ s^−1^ (Table S4; Fig. 4). *C. canescens* is most abundant in the least wet habitats, mainly on tops of sedge tussocks, in our case those of *C. acuta*. Competition is decisive for the balance between *C. acuta* and *C. canescens*, being influenced mainly by the water régime^65^. *C. canescens* thrives in the terrestrial ecophase while *C. acuta* takes advantage of the limosal ecophase^59^. In wet years, *C. acuta* prevailed in the stand whereas *C. canescens* prevailed mainly in dry years^65^.

*Phalaris arundinacea* is a grass forming communities occupying habitats along stream and river banks^62^. In the Wet Meadows, *P. arundinacea* occupies the nutrient-rich banks of the Central Channel and forms either monodominant stands or stands in which it is mixed with the other plant species studied. The P_N_/I relationship of *P. arundinacea* itself attains a lower A_sat_ (9.411 μmol m^−2^ s^−1^) than *C. acuta* (A_sat_ = 10.825 μmol m^−2^ s^−1^). The importance of *P. arundinacea* is relatively greatest in the spring period, when its stems and leaves expand early, together with those of *A. calamus,* and are photosynthetically highly active. *P. arundinacea* thus contributes significantly to the photosynthesis of the “big-leaf” mainly during the spring period. *G. maxima* also plays an important role at this time, thanks to its rate of photosynthesis reaching its seasonal maximum in spring.

We can conclude that the real plant species composition is mixed at the study site suitably for reaching an optimum physiological state of the local vegetation for its rapid growth and a high P_N_/I relationship. The growth optima of the studied plant species are different, these differences being evident also in their different P_N_/I relationships (Fig. 5). Ecological niches of these plant species are therefore also different and overlap only partly. The relatively poorer growth of the sedges is competitively disadvantageous under the “dry” conditions of a long-lasting terrestrial ecophase^59,66^. In reality, a drop of their photosynthesis under the “dry” conditions, associated with low A_sat_ values, can probably be compensated by the photosynthesis of other plant species, especially non-wetland ones. These plants tend to invade the Wet Meadows site during a long-lasting terrestrial ecophase. The effect of invasions was not an object of the present study, but it may represent a challenging task for future studies.

## Conclusions

The parameters of the P_N_/I relationship significantly differed among all five plant species studied. These differences indicate that ecological niches of these species are different and overlap only partly. The “big-leaf” concept used for upscaling the parameters obtained for individual species to the scale of both partial and overall growth periods (with respect to the whole growing season) was very convenient and useful. Differences in the P_N_/I relationship among the growth periods can be related to structural differences among individual species and also to the keystone role of *C. acuta* in the spatial structure of the whole plant community. Species composition (diversity) is essential for reaching a light-saturated rate of photosynthesis (A_sat_) of the “big-leaf” which represents the sedge-grass marsh in different growth periods. In this respect, our study is leaving open the question of the effect on the P_N_/I parameters of the sedge-grass marsh as a “big-leaf” of non-wetland plants spontaneously invading the Wet Meadows site.

All parameters of the P_N_/I relationship of the individual plant species studied and individual growth periods are summarized in the Supplementary Information (Tables S3, S4 and S5) for possible use in growth and carbon sequestration models based on the “big-leaf” approach to modelling canopy carbon fluxes. We hope that these parameters can be widely used in this way in both current and future studies.

## Supplementary Information

Supplementary Informations.

## Data Availability

The datasets from in situ measurements of the current study are available from the corresponding author on reasonable request.
