# Supplementary material for: Photosynthetic parameters of a sedge-grass marsh as a big-leaf: effect of plant species composition
Source: Sci Rep. 2021 Feb 12;11:3723. doi: 10.1038/s41598-021-82382-2 (PMC7881001; doi:10.1038/s41598-021-82382-2)
Supplement: Supplementary file 1 — Supplementary Informations. [file 41598_2021_82382_MOESM1_ESM.pdf]

Supplementary Information
Scientific Reports

Photosynthetic parameters of a sedge-grass marsh as a "big-leaf": effect of plant species composition

Markéta Mejdová[1, 2], Jiří Dušek[1], Lenka Foltýnová[1], Lenka Macálková[1], Hana Čížková[2]

[1]Global Change Research Institute AS CR, v. v. i., Bělidla 98/4a, Brno, Czech Republic

[2]Faculty of Agriculture, University of South Bohemia, Studentská 1668, CZ-370 05 České Budějovice, Czech Republic

Corresponding author: Markéta Mejdová (marketahonissova@gmail.com)

Selection of light response curves

Many mathematical models can be used to describe photosynthesis-irradiance (PN/I) curves. In our study we selected four types of mathematical models that can be used for detailed evaluation of the PN/I relationship. The used models with their mathematical equations are shown in Table 1. The best model of PN/I curve was chosen on the basis of the lowest values of the following statistical parameters: Mean square error (MSE) (Eq. 1), Root mean square error (RMSE) (Eq. 2), Relative root mean square error (RRMSE) (Eq. 3), Mean absolute error (MAE) (Eq. 4) and the highest value of Effectivity (EF) (Eq. 5) and also by the value of BIAS (Eq. 6). Tests of these statistical criteria were used for choosing a suitably fitting model. The result would be unreliable if only one or very few criteria were taken into account. In most situations the best model had the best values of all the criteria except one or two. The use of all criteria eliminated a bad model selection. The data were evaluated using the R statistical software version 3.6.11.

Mean square error $$MSE = \frac{1}{n}\sum_{i=1}^{n}(o_i - p_i)^2 \quad (1)$$

Root mean square error $$RMSE = \sqrt{MSE} \quad (2)$$

Relative root mean square error $$RRMSE = \frac{RMSE}{\acute{o}} \quad (3)$$

Mean absolute error $$MAE = \frac{1}{n}\sum_{i=1}^{n}|o_i - p_i| \quad (4)$$

Effectivity $$EF = 1 - \frac{\sum_{i=1}^{n}(o_i - p_i)^2}{\sum_{i=1}^{n}(o_i - \acute{o})^2} \quad (5)$$

BIAS $$bias = \acute{o} - \acute{p} \quad (6)$$

where o_i are the observed values, pi is fitted values, n is the number of observations, o ´ is the mean of observed values and p ´ is the mean of fitted values. Non-rectangular hyperbola (model 4 in Table 1) was selected as the best fitting overall model. according to all criteria tested in most situations (Table 2). The use of the nonrectangular hyperbola model is not restricted to measurements of photosynthesis on the leaf scale. The hyperbolic light-response model was also used as a model of the response of the net ecosystem exchange (NEE) to light[2,3]. This response model is known as the rectangular hyperbola or the Michaelis–Menten type model[4,5]. The NEE response to amount of incident PAR is similar to the light response of leaves, but the response of NEE (in agreement with the definitions of NEE[6,7]; is considered as the response of a whole ecosystem. The light response of the NEE is often used for determining the partitioning of NEE into GPP and RE[8,9].

Table S1 Overview of the models with equations used for fitting the response of photosynthesis to the amount of incident PPFD. The sources of individual models are also given. The original source of the equation is highlighted by bold letters.

**Model/** Equation	Source
**Rectangular hyperbola**<br><br>(1) $Pn = \frac{\alpha * I * P_{\max}}{\alpha * I + P_{\max}} - R_d$	**Baly**[1]**,** Kyei-Boahen et al.[2],<br><br>Lobo et al.[3]
(2) $Pn = \frac{\alpha * I * P_{\max}}{\sqrt{\alpha^2 * I^2 + P_{\max}^2}} - R_d$	**Smith**[4] **,** Lobo et al.[3]
**Exponential model**<br><br>(3) $Pn = P_{\max}\left[1 - \exp\left(\frac{-\alpha * I}{P_{\max}}\right)\right] - R_d$	**Webb et al.**[5] **,** Steele[6], Lewis and Smith[7], Ralph and Gademann[8] ,Lin et al.[9], Lobo et al.[3]
**Nonrectangular hyperbola**<br><br>(4)<br><br>$Pn = \frac{\alpha * I + P_{\max} - \sqrt{(\alpha * I + P_{\max})^2 - 4\theta * \alpha * I * P_{\max}}}{2\theta} - R_d$	**Marshall and Biscoe** [10]**,** Leverenz [11–13], Li et al.[14], Araus et al.[15], Ögren[16], Ögren and Evans[17],Thornley[18], Marschall and Proctor[19], Wu et al.[20]; Jones[21], Lieth and Pasian[22], Sorrell et al.[23], Lobo et al.[3], Xu et al. [24]
**Ye model**<br><br>(5) $Pn = \alpha_{(I - I_{comp})} * \frac{1 - \beta * I}{1 + \gamma * I} * (I - I_{comp}) - R_d$	**Ye**[25]**,** Lobo et al.[3]

P - the rate of photosynthesis [μmol (CO_2) m^{-2} s^{-1}]
A_{sat} - light-saturated photosynthesis [μmol (CO_2) m^{-2} s^{-1}]
I - irradiance, photosynthetic photon flux density PPFD [μmol (photon) m^{-2} s^{-1}]
I_{comp} - light compensation point [μmol (photon) m^{-2} s^{-1}]
α - maximum quantum yield [μmol (CO_2) μmol (photon)$^{-1}$]
θ - convexity (dimensionless)
k – adjusting factor [s m^2 μmol (photon)$^{-1}$]
β - adjusting factor (dimensionless)
γ- adjusting factor (dimensionless)
R_d – respiration under dark conditions [μmol (CO_2) m^{-2} s^{-1}]

Table S2 Results of the selection procedure for optimal response curves for the plant species studied. Equations of the models are presented in Table 1. Data on the frequencies of occurrence of individual models of fitting the photosynthetic amount of incident PAR curves and total frequency sums for the five species studied (Ph - *Phalaris arundinacea*, Car - *Carex acuta*, Gl – *Glyceria maxima*, Ac - *Acorus calamus*, Cal - *Calamagrostis canescens*) during the 2013 growing season. The best model is indicated in bold types.

Model	Frequency of significance for individual fitting models					
	Plant species					Total
	Ph	*Car*	*Gl*	*Ac*	*Cal*	
Rectangular hyperbola (1)	0	0	1	1	0	2
Rectangular hyperbola (2)	1	0	1	2	2	6
Exponential model (3)	5	1	2	0	2	10
**Nonrectangular hyperbola (4)**	**5**	**6**	**7**	**9**	**5**	**32**
Ye model (5)	3	6	3	1	2	15

Table S3 Means, standard errors of means (SE) and medians of photosynthetic light curve parameters for the dominant plant species during the growing season. Apparent maximum quantum yield (α), light-saturated photosynthesis (A_{sat}), light-compensation point (I_{comp}) and dark respiration (R_d), dimensionless convexity (θ), number of measurements (n).

Curve parameters		α [$\mu mol\ CO_2\ \mu mol\ (photon)^{-1}$]		A_{sat} [$\mu mol\ CO_2\ m^{-2}\ s^{-1}$]		I_{comp} [$\mu mol\ (photon)\ m^{-2}\ s^{-1}$]		R_d [$\mu mol\ CO_2\ m^{-2}\ s^{-1}$]		Θ [dimensionless]	
Plant species	n	Mean ± SE	Median	Mean ± SE	Median	Mean ± SE	Median	Mean ± SE	Median	Mean ± SE	Median
P. arundinacea	15	0.073 ± 0.010	0.073	9.411 ± 0.885	9.310	27.155 ± 5.978	18.062	-1.448 ± 0.438	-0.965	0.559 ± 0.111	0.819
C. acuta	15	0.041 ± 0.006	0.037	10.825 ± 0.783	9.310	23.899 ± 5.210	19.738	-0.716 ± 0.115	-0.695	0.217 ± 0.082	0.051
A. calamus	13	0.069 ± 0.008	0.065	16.605 ± 1.508	17.931	35.080 ± 6.539	29.517	-2.275 ± 0.533	-1.630	0.352 ±0.092	0.239
C. canescens	11	0.054 ± 0.011	0.043	7.0219 ± 1.285	5.517	23.591 ± 6.342	22.489	-0.844 ± 0.214	-0.660	0.403 ± 0.406	0.171
G. maxima	14	0.078 ± 0.006	0.073	18.362 ± 1.327	17.50	13.485 ± 3.273	14.253	-1.079 ± 0.273	-1.080	0.195 ± 0.066	0.051

Table S4 Means of photosynthetic light-curve parameters of the individual growth periods and whole growing season under measured real conditions, hypothetical wet and dry conditions: apparent maximum quantum yield (α), light-saturated photosynthesis (A_{sat}), irradiance compensation point (I_{comp}) and dark respiration (R_d), Values are weighted by the plant species´ partial LAI values.

Curve parameters		Situations											
		Measured real conditions				Assumed wet conditions				Assumed dry conditions			
symbol	unit	Spring	Summer	Autumn	Season	Spring	Summer	Autumn	Season	Spring	Summer	Autumn	Season
α	[$\mu mol\ CO_2\ \mu mol\ (photon)^{-1}$]	0.0484	0.0548	0.0487	0.065	0.0534	0.0500	0.0659	0.0606	0.0405	0.0677	0.0489	0.0602
A_{sat}	[$\mu mol\ CO_2\ m^{-2}\ s^{-1}$]	10.903	7.950	5.994	12.666	12.853	9.621	8.793	13.270	5.457	7.862	6.905	9.0792
I_{comp}	[$\mu mol\ (photon)\ m^{-2}\ s^{-1}$]	15.896	23.488	13.994	13.485	16.313	25.799	1.069	13.485	14.571	17.583	13.479	13.485
R_d	[$\mu mol\ CO_2\ m^{-2}\ s^{-1}$]	-0.692	-1.134	-0.702	-1.2	-0.772	-1.098	-0.834	-1.147	-0.478	-1.000	-0.667	-1.047

Table S5 Significance (p-values) of tested differences between the dominant plant species (Ph - *P. arundinacea*, Car - *C. acuta*, Gl - *G. maxima*, Ac - *A. calamus*, Cal - *C. canescens*) in their: apparent maximum quantum yield (α), light-saturated photosynthesis (A_{sat}), light-compensation point (I_{comp}) and dark respiration (R_d). The differences were tested using the Kruskal-Wallis test, post-hoc testing was performed by the Dunn test with Bonferroni correction for multiple comparison. P-values of the Kruskal-Wallis test for each variable are listed in the last line of the table. The P-values are adjusted, so the zero hypothesis is rejected when $p < 0.05$.

Plant species	DF	α	Asat	I_{comp}	R_d
Ph & Car	13	0.11	1.00	1.00	1.00
Ph & Gl	12	1.00	**0.001**	1.00	1.00
Ph & Ac	12	1.00	**0.012**	1.00	1.00
Ph & Cal	11	1.00	1.00	1.00	1.00
Car & Gl	11	**0.02**	**0.010**	1.00	1.00
Car & Ac	12	0.20	0.112	1.00	0.07
Car& Cal	9	1.00	0.98	1.00	1.00
Gl & Ac	12	1.00	1.00	0.12	1.00
Gl & Cal	10	0.50	**$<10^{-3}$**	1.00	1.00
Ac & Cal	10	1.00	**0.001**	1.00	0.22
K-W test	-	**0.01**	**$<10^{-3}$**	0.30	0.06

Supplementary References

1. Baly, E. C. The kinetics of photosynthesis. Proceedings of the Royal Society of London. Series B - Biological Sciences **117**, 218–239 (1935).
2. Kyei-Boahen, S., Lada, R., Astatkie, T., Gordon, R. & Caldwell, C. Photosynthetic Response of Carrots to Varying Irradiances. Photosynthetica **41**, 301–305 (2003).
3. Lobo, F. de A. et al. Fitting net photosynthetic light-response curves with Microsoft Excel — a critical look at the models. Photosynthetica **51**, 445–456 (2013).
4. Smith, E. L. Photosynthesis in relation to light and carbon dioxide. Proceedings, National academy of sciences (USA) **22**, 504–511 (1936).
5. Webb, B. Trends in stream and river temperature. HYDROLOGICAL PROCESSES **10**, 205–226 (1996).
6. Steele, J. H. Environmental Control of Photosynthesis in the Sea. Limnology and Oceanography **7**, 137–150 (1962).
7. Lewis, M. & Smith, J. A small volume, short-incubation-time method for measurement of photosynthesis as a function of incident irradiance. Marine Ecology Progress Series **13**, 99–102 (1983).
8. Ralph, P. J. & Gademann, R. Rapid light curves: A powerful tool to assess photosynthetic activity. Aquatic Botany **82**, 222–237 (2005).
9. Lin, S.-T., Guan, B. T. & Chang, T.-Y. Fitting Photosynthesis Irradiance Response Curves with Nonlinear Mixed-effects Models. 15 (2008).
10. Marshall, B. & Biscoe, P. V. A Model for C3 Leaves Describing the Dependence of Net Photosynthesis on Irradiance. Journal of Experimental Botany **31**, 29–39 (1980).
11. Leverenz, J. W. & Jarvis, P. G. Photosynthesis in Sitka Spruce. VIII. The Effects of Light Flux Density and Direction on the Rate of Net Photosynthesis and the Stomatal Conductance of Needles. The Journal of Applied Ecology **16**, 919–932 (1979).
12. Leverenz, J. W., Falk, S., Pilstrm, C.-M. & Samuelsson, G. The effects of photoinhibition on the photosynthetic light-response curve of green plant cells (Chlamydomonas reinhardtil). Planta **1990**, 161–168 (1990).
13. Leverenz, J. W. Shade shoot structure of conifers and the photosynthetic response to light at two CO2 partial pressures. Functional Ecology **9**, 413–421 (1995).
14. Li, M., Hou, G., Yang, D., Deng, G. & Li, W. Photosynthetic traits of Carex cinerascens in flooded and nonflooded conditions. Photosynthetica **48**, 370–376 (2010).

15. Araus, J. L., Alegre, L., Tapia, L., Calafell, R. & Serret, M. D. Relationships between photosynthetic capacity and leaf structure in several shade plants. American Journal of Botany **73**, 1760–1770 (1986).
16. Ogren, E. Convexity of the Photosynthetic Light-Response Curve in Relation to lntensity and Direction of Light during Growth'. **101**, 7 (1993).
17. Ogren, E. & Evans, J. R. Photosynthetic light-response curves: I. The influence of CO2 partial pressure and leaf inversion. Planta **189**, (1993).
18. Thornley, J. Dynamic Model of Leaf Photosynthesis with Acclimation to Light and Nitrogen. Annals of Botany **81**, 421–430 (1998).
19. Marschall, M. & Proctor, M. C. F. Are Bryophytes Shade Plants? Photosynthetic Light Responses and Proportions of Chlorophyll a, Chlorophyll b and Total Carotenoids. Annals of Botany **94**, 593–603 (2004).
20. Wu, J. et al. Photosynthetic characteristics of dominant tree species and canopy in the broadleaved Korean pine forest of Changbai Mountains. Science in China Series D: Earth Sciences **49**, 89–98 (2006).
21. Jones, M. B. Photosynthetic Responses of C 3 and C 4 Wetland Species in a Tropical Swamp. The Journal of Ecology **76**, 253 (1988).
22. Lieth, J. H. & Pasian, C. C. A Model for Net Photosynthesis of Rose Leaves as a Function of Photosynthetically Active Radiation, Leaf Temperature, and Leaf Age. Journal of the American Society for Horticultural Science **115**, 486–491 (1990).
23. Sorrell, B. K., Brix, H., Fitridge, I., Konnerup, D. & Lambertini, C. Gas exchange and growth responses to nutrient enrichment in invasive Glyceria maxima and native New Zealand Carex species. Aquatic Botany **103**, 37–47 (2012).
24. Xu, J. et al. A general non-rectangular hyperbola equation for photosynthetic light response curve of rice at various leaf ages. Scientific Reports **9**, (2019).
25. Ye, Z.-P. A new model for relationship between irradiance and the rate of photosynthesis in Oryza sativa. Photosynthetica **45**, 637–640 (2007).
